# Dying to be Thin: The complex relationship between Eating Disorders, Epilepsy and Treatment Resistant Depression

**DOI:** 10.1192/j.eurpsy.2025.1396

**Published:** 2025-08-26

**Authors:** D. J. K. Fones, H. Y. Lee

**Affiliations:** 1 Psychiatry, Singapore General Hospital, Singapore, Singapore

## Abstract

**Introduction:**

Depression is a common comorbidity in patients with eating disorders. Epilepsy significantly impacts mood and personality, with up to one-third of epilepsy patients experiencing psychiatric comorbidities. The coexistence of eating disorders, epilepsy, and depression presents a clinical challenge due to complex neurological, psychiatric, and psychosocial interactions. Despite well-established links between these conditions, little literature explores their convergence in a single patient.

**Objectives:**

We describe a case of anorexia nervosa (AN) in a premorbidly well woman who subsequently developed idiopathic generalized epilepsy. She then developed depression and an intractable urge to end her life solely because she felt “fat”. We discuss the complex relationship between these conditions and propose hypotheses that may explain this interaction.

**Methods:**

Informed consent was obtained from Ms. O to access her medical records for this case report. We reviewed her medical history, psychiatric evaluations and treatment interventions.

**Results:**

Miss O is a 24-year old ex-nursing student with no past psychiatric history and was described as a bubbly young girl. She first presented in Nov 2018 with AN (BMI 14.0) achieving weight restoration by Aug 2019 after developing binge-eating episodes. In Jan 2020, she was diagnosed with epilepsy. Shortly thereafter, she developed severe depression, accompanied by personality changes, self-harm behaviours, and intractable suicidal ideations. She attributed her suicidality to her body image disturbances and perceived weight gain. She continues to restrict and purge but her weight has stabilized around BMI 20-22. Since then, she has had 13 admissions for suicide attempts and 7 for managing depression. Her treatments included antidepressants, mood stabilizers, antipsychotics, electroconvulsive therapy, repetitive transcranial magnetic stimulation and intravenous ketamine but her condition remained treatment-resistant.

We propose several hypotheses to explore the interactions between AN, epilepsy, and treatment-resistant depression. These include hypothalamic-pituitary-adrenal axis dysregulation and neuroinflammation, potential common neurological pathways between AN and epilepsy, the possible development of personality disorders, and cognitive distortions where disordered eating and suicidal behaviours serve as maladaptive control mechanisms. We also explored concepts like epileptic personality, interictal dysphoric disorder, and the interplay between antiepileptic drugs and mood.

**Conclusions:**

These hypotheses collectively highlight the complex mechanisms that likely underlie Ms. O’s comorbid AN, epilepsy, and treatment-resistant depression emphasizing the need for integrated, multidimensional treatment approaches. Further research is essential to develop targeted interventions for such challenging comorbidities.

**Disclosure of Interest:**

None Declared

